# The multidimensional prognostic index in hospitalized older adults: practicability with regard to time needs

**DOI:** 10.1007/s40520-022-02311-9

**Published:** 2023-01-30

**Authors:** Selma Irmgard Bonnekoh, Anna Maria Meyer, Lena Pickert, Ralf-Joachim Schulz, Ingrid Becker, M. Cristina Polidori

**Affiliations:** 1 Ageing Clinical Research, Department II of Internal Medicine and Center for Molecular Medicine Cologne, Faculty of Medicine and University Hospital Cologne, Cologne, Germany; 2 Clinic of Geriatrics, St. Marien Hospital Cologne, Cologne, Germany; 3 Institute of Medical Statistics and Computational Biology, Faculty of Medicine and University Hospital of Cologne, Cologne, Germany; 4 CECAD Research Center, Ageing Clinical Research, Cologne, Germany

**Keywords:** Multidimensional prognostic index, Time resources, Applied health science, Comprehensive geriatric assessment, Health economics, Frailty

## Abstract

**Background:**

Comprehensive Geriatric Assessment (CGA) is decisive in patient-centered medicine of the aged individual, yet it is not systematically used.

**Aim:**

The aim of this study was to provide precise practice-relevant time expenditure data for the Multidimensional Prognostic Index (MPI), a questionnaire-based frailty assessment tool.

**Methods:**

MPI was determined in ninety older multimorbid adults in three geriatric departments (cohorts 1, 2 and 3). The time needed to perform the MPI (tnpMPI) was recorded in minutes. Follow-up data were collected after 6 months.

**Results:**

The median tnpMPI was 15.0 min (IQR 7.0) in the total collective. In the last visited cohort 3, the median was 10.0 min and differed significantly from cohorts 1 and 2 with medians of 15.5 and 15.0 (*p* < 0.001).

**Conclusion:**

These findings indicate, that MPI, as a highly informative frailty tool of individualized medicine, can be performed in an adequately practicable time frame.

**Supplementary Information:**

The online version contains supplementary material available at 10.1007/s40520-022-02311-9.

## Introduction

Demographic changes challenge health care systems with regard to high needs in efficacious geriatric patient care [1]. Comprehensive Geriatric Assessment (CGA) is considered as a valuable diagnostic tool for patient-centered care in older persons and may enable prevention of negative outcomes [2]. In the frame of CGA, a plethora of assessment tools has been validated and introduced in national and international settings [3]. Nevertheless, none of these emerged as a regularly applied and overall accepted so-called gold standard up to now [4]. The barriers in establishing CGA in everyday clinical care are complex. They include individual patient- and health care professional-related factors as well as lack of social, political and legal substantiation [5]. Increasingly considered as an important contributory factor is the absence of sufficient study-based data investigating practicability and feasibility of CGA under real world conditions [6]. In this context, the necessity of applied health research and implementation science, both in parallel to basic clinical research, becomes apparent [7].

The present study aims at measuring the exact time expenditure to carry out the Multidimensional Prognostic Index (MPI), an accurate, highly validated CGA-based prognostic and frailty tool [8–10].

## Methods

### Patients and assessment

**Fig. 1:**
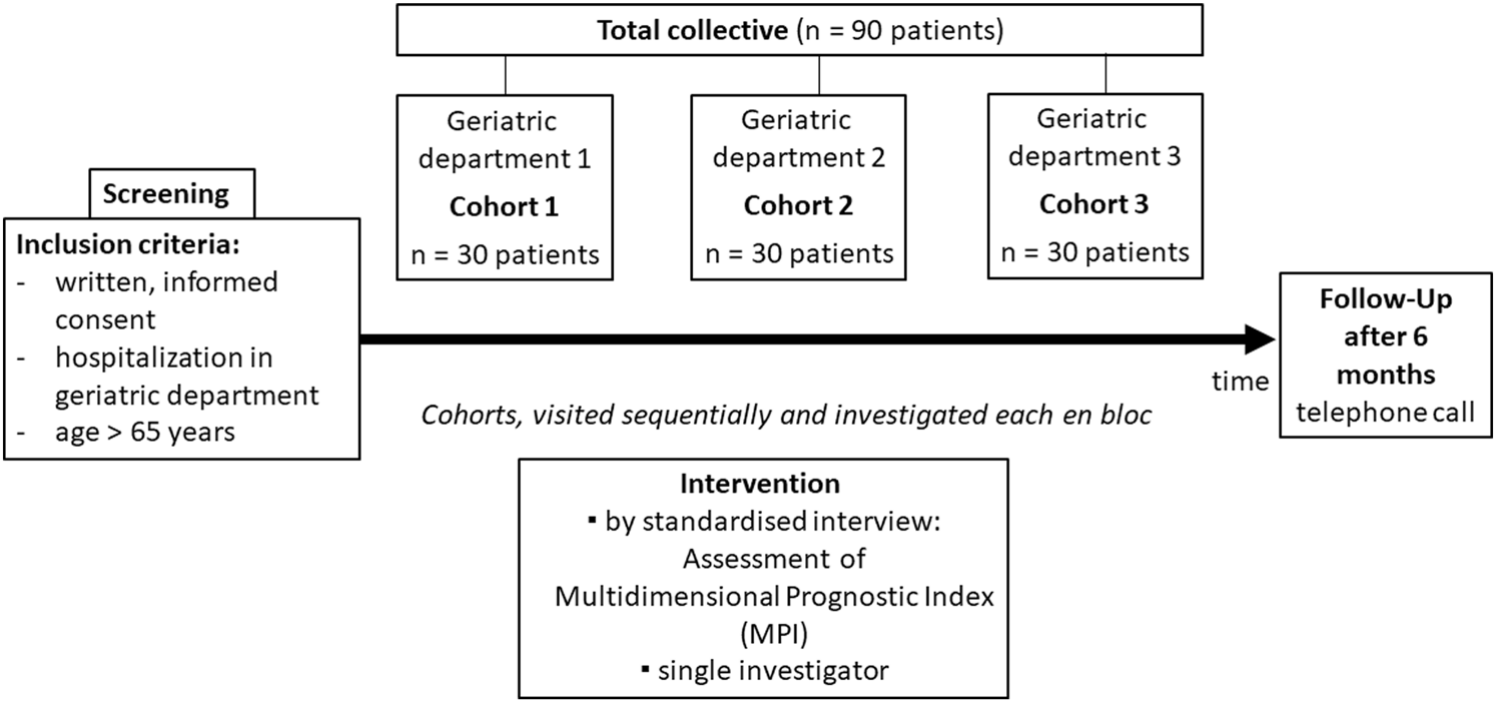
Flowchart of the study—design of the feasibility study investigating the time requirements for the performance of the Multidimensional Prognostic Index (MPI)

The study was approved by the Ethics Committee of the University of Cologne (EK 17-101) and registered accordingly (DRKS00017071). Inclusion criteria were an age older than 65 years, multimorbidity (i.e., more than two chronic conditions requiring long-term treatment), hospitalization in the geriatric unit and providing informed consent. Reason for exclusion was inability to give consent (e.g., by decisional impairment in case of advanced dementia). MPI data were collected by 1 investigator in 90 older multimorbid patients hospitalized between September 2017 and October 2019. Three geriatric departments of hospitals in Cologne, Germany participated in the study: Malteser Krankenhaus St. Hildegardis (cohort 1); Evangelisches Krankenhaus Kalk (cohort 2); St. Marien Hospital (cohort 3). In the aforementioned sequel, the three participating departments were visited sequentially. A cohort of 30 patients, each, was investigated en bloc as shown in the flowchart of the study (Fig. 1). After signing informed consent, patients underwent a structured evaluation including the eight domains of co-habitation status, number of drugs taken, functions (Activities of Daily Living (ADL), Instrumental Activities of Daily Living (IADL)), cognition (Short Portable Mental Status Questionnaire (SPMSQ)), pressure ulcer risk (Exton-Smith-Scale (ESS)), multimorbidity (Cumulative Index Rating Scale (CIRS)), and nutritional status (Mini Nutritional Assessment—Short Form (MNA-SF)) as previously described [10, 11]. The scores were included in a mathematical algorithm delivering the MPI and its three mortality risk subgroups: low (MPI 1: 0.00–0.33), moderate (MPI 2: 0.34–0.66) and severe risk (MPI 3: 0.67–1.0) [10]. During the assessment, the time expenditure to collect the scores for all MPI domains and subsequently to calculate the MPI-score was recorded: time needed to perform MPI (tnpMPI) in minutes (min). An assessment time that was considered feasible in clinical practice was not specified before data collection and analysis were undertaken, in terms of a hypothesis-free approach. Additionally, demographics (age, gender) and health-related data (individual need of care, admission date and admission diagnosis) were recorded. The main objective of the study was to assess the time needed to perform MPI (tnpMPI). Since the MPI is a prognostic tool predicting mortality as described before [8], we performed a follow-up as a descriptive, secondary objective. Rehospitalisations, individual need of care, falls, medication and mortality were assessed by a telephone call at 6 months after the initial interview.

### Statistical analysis

Descriptive statistics are presented using absolute numbers and relative frequencies for categorical variables. Medians (interquartile range, IQR) were used for continuous and ordinal variables. Spearman correlation was applied for statistical analysis of dependence between tnpMPI and other relevant variables. Kruskal–Wallis-test was performed for comparison of medians. In case of statistical significance, Mann–Whitney-*U*-test with Bonferroni adjustment was employed for post hoc analysis. Frequencies were compared with the Chi-square test. The overall significance level *α* was set to 0.05. All statistical analyses were performed using SPSS (Statistical Package for Social Sciences, SPSS Inc., Chicago, IL, version 27.0).

## Results

### Demographics

Demographic and clinical characteristics of study participants according to MPI group are shown in Table 1. Of the *n* = 90 recruited study participants, 65 were women (72%, Table 1). The median age was 83.5 years (IQR 7.0, Table 1). Median MPI was 0.38 (IQR 0.25). The majority of participants belonged to MPI risk group 2 (62%), followed by risk group 1 (32%) and risk group 3 (6%, Table 1). The MPI risk group 1 patients were significantly younger than patients in risk group 2 (*p* = 0.03, Table 1). The main diagnoses for hospital admission were post-traumatic condition (34%), immobility and pneumonia (7%, each), heart failure, as well as gastrointestinal diseases and vertigo / fall (6%, each). Overall, the number of diseases was at a median of 6 (IQR 5.0, Table 1). Almost 55% of the study participants took 6 or more drugs per day and met the criteria for polypharmacy, with a median of 6 drugs per patient (IQR 5.0–8.0, Table 1).

**Table 1:** Analysis of study population according to Multidimensional Prognostic Index (MPI) risk groups; distribution of gender, age and diseases (descriptive statistics)

Condition	Total *n* = 90	MPI 1 *n* = 29 (32.2%)	MPI 2 *n* = 56 (62.2%)	MPI 3 *n* = 5 (5.6%)	*p* value	*post-hoc-*test
Gender, female [vs. male]; *n* (%)	65 (72.2)	23 (79.3)	37 (66.1)	5 (100.0)	0.160	n.d
Age (years), median (IQR)	83.5 (7.0)	82.0 (6.0)	85.0 (7.0)	84.0 (5.0)	0.030*	MPI 1 < 2
Main admission diagnosis, *n* (%)						
Post-traumatic condition	31 (34)	13 (44.8)	18 (32.1)	0 (0)	n.d	n.d
Immobility	6 (7)	4 (13.8)	1 (1.8)	1 (20.0)	n.d	n.d
Pneumonia	6 (7)	1 (3.4)	5 (8.9)	0 (0)	n.d	n.d
Heart failure	5 (6)	1 (3.4)	3 (5.4)	1 (20.0)	n.d	n.d
Gastrointestinal disease	5 (6)	0 (0)	5 (8.9)	0 (0)	n.d	n.d
Vertigo/fall	5 (6)	1 (3.4)	3 (5.4)	1 (20.0)	n.d	n.d
Number of diagnoses, median (IQR)	6 (4–9)	5 (3–6)	7 (5–9)	6 (4–8.5)	0.003*	MPI 1 < 2
Polypharmacy, *n* (%)	49 (54.4)	10 (34.4)	35 (62.5)	4 (80.0)	n.d	n.d
M-MPI domains, median (IQR):						
CIRS	1.0 (1.0–2.0)	1.0 (0.0–2.0)	2.0 (1.0–3.0)	2.0 (1.0–2.5)	0.008*	MPI 1 < 2
ADL	5.0 (3.0–5.0)	6.0 (5.0–6.0)	4.5 (3.0–5.0)	1.0 (0.5–2.0)	< 0.001*	MPI 1 > 2 > 3
IADL	5.0 (3.0–6.0)	7.0 (6.0–7.5)	5.0 (3.0–5.0)	1.0 (1.0–2.0)	< 0.001*	MPI 1 > 2 > 3
MNA-SF	6.0 (6.0–8.3)	7.0 (6.0–9.0)	6.0 (5.35–8.0)	6.0 (6.0–9.0)	0.695	n.d
SPMSQ	0.0 (0.0–1.0)	0.0 (0.0–0.0)	0.0 (0.0–1.0)	1.0 (0.5–3.0)	0.003*	MPI 1 < 3
ESS	16.0 (14.0–17.0)	16.0 (16.0–17.0)	15.0 (14.0–17.0)	13.0 (11.0–15.0)	0.001*	MPI 1 > 2 MPI 1 > 3
Number of drugs	6.2 (5.0–8.0)	4.8 (4.0–6.5)	6.8 (5.0–8.0)	7.6 (6.0–9.5)	0.003*	MPI 1 < 2 MPI 1 < 3
Cohabitation status, *n* (%):					0.002*	MPI 1 < 2
With family members	30 (33.3)	16 (55.2)	14 (25.0)	0 (0.0)		
Professional care	7 (7.8)	3 (10.3)	4 (7.1)	0 (0.0)		
Alone	53 (58.9)	10 (34.5)	38 (67.9)	5 (100.0)		

Total collective of patients comprising cohorts 1 to 3, each visited *en bloc* sequentially in three geriatric units; Kruskal–Wallis-test, level of significance < 0.05

*n* number of patients, *IQR* inter quartile range, *CIRS* cumulative index rating scale, *ADL* activities of daily living, *IADL* instrumental activities of daily living, *MNA-SF* mini nutritional assessment—short form, *SPMSQ* short portable mental status questionnaire, *ESS* exton-smith-scale, *n.d.* not done

*statistically significant

### Primary outcome: tnpMPI

**Fig. 2:**
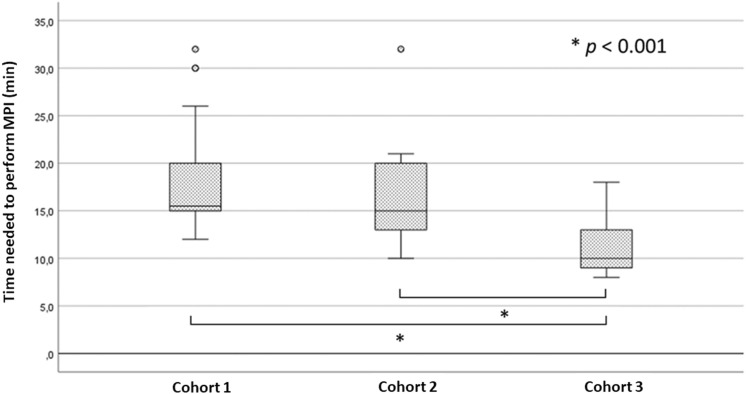
Time needed to perform MPI (min) in the three geriatric departments (cohorts 1–3) as visited en bloc sequentially

In the overall patient sample, the median tnpMPI was 15 min (IQR 7.0) with a minimum of 8 min (Min) and a maximum of 32 min (Max, Table 2). Comparison of the tnpMPI in cohorts 1, 2 and 3, visited consecutively, resulted in a highly significant minimum median tnpMPI of 10.0 min (IQR 4.3) for cohort 3, decreasing from 15.5 min (median, IQR 5.3) to 15.0 min (median, IQR 7.0) for cohorts 1 and 2 (*p* < 0.001 each, post hoc test, Fig. 2). The tnpMPI in cohort 3 showed 75 and 85 percentiles of 13 min and 15 min, respectively (Table 2).

**Table 2:** Time expenditure for the performance of Multidimensional Prognostic Index (MPI) in the total collective and in the cohorts 1 to 3

	Time needed to perform MPI in minutes (min)			
	Total collective *n* = 90	Cohort 1 *n* = 30	Cohort 2 *n* = 30	Cohort 3 *n* = 30
Median	15.0	15.5	15.0	10.0*
IQR	7.0 (11.0–18.0)	5.3 (15.0–20.3)	7.0 (13.0–20.0)	4.3 (8.7–13.0)
Range (min–max)	8.0–32.0	12.0–32.0	10.0–32.0	8.0–18.0
Percentile				
25	11.0	15.0	13.0	8.7
50	15.0	15.5	15.0	10.0
75	18.0	20.3	20.0	13.0
80	19.0	21.8	20.0	13.0
85	20.0	25.4	20.0	15.0
90	21.0	29.6	20.9	16.8
95	27.8	30.9	26.0	18.0
100	32.0	32.0	32.0	18.0

Total collective of patients comprising cohorts 1 to 3, each visited *en bloc* sequentially in three geriatric departments

*N* number of patients; range from minimum to maximum

*cohort 3 differing significantly from cohorts 1 and 2, each in post hoc test (*p* < 0.05)

A significant negative correlation was found between tnpMPI and the date of the patients’ interview (*r* = − 0.559; *p* < 0.01, Table 3), as well as between tnpMPI and the cohort assignment (*r* = − 0.608; *p* < 0.01, Table 3). There was no correlation between tnpMPI and age, gender, MPI value, SPMSQ, CIRS or polypharmacy (Table 3).

**Table 3:** Dependence between time needed to perform Multidimensional Prognostic Index (tnpMPI) and selected variables

	*r*	*p* value
Age	0.420	0.691
Gender	− 0.710	0.506
MPI value	− 0.168	0.113
Assessment date	− 0.559	< 0.01*
Cohort assignment	− 0.608	< 0.01*
SPMSQ^#^	0.132	0.215
CIRS	− 0.159	0.135
Polypharmacy	− 0.153	0.150

*tnpMPI* time needed to perform MPI, *r* Spearman correlation coefficient, *SPMSQ*^*#*^ short portable mental status questionnaire (categorized for mild, moderate and severe mental impairment), *CIRS* cumulative index rating scale

*tnpMPI correlating negatively with assessment date and cohort assignment

*Statistically significant

Regarding the aforementioned cohort-dependence of tnpMPI, it was investigated whether the three cohorts differed in terms of the parameters age, gender, number of diagnoses, number of medications, polypharmacy and the MPI domains (see Supplementary Table 1). A significant difference was found only for the number of diagnoses, which was significantly higher in cohort 3 with a median of 8 diagnoses compared to 6 and 4 in cohorts 1 and 2, respectively (*p* < 0.004, Supplementary Table 1).

### Six months’ follow-up

Of 90 study participants, 57 were followed-up at 6 months (loss to follow-up rate of 40%). Death was reported in one case. Statistical analysis did not reveal association between MPI value and rehospitalisation, falls, grade of care or medication. The high rate of loss to follow-up did not allow statistical analysis of mortality or survival.

## Discussion

This pragmatic investigation shows that the CGA-based MPI with high information output can be evaluated, in a complex real-life geriatric population, in as low as 15.0 min (as median) across all sites. This corresponds approximately to an average duration of “ < 15 min”, as estimated in a meta-analysis comparing various CGA tools by Dent et al. (2016) [4]. Noteworthy, the MPI, with its eight domains and 51 items, is certainly a highly complex representative of the available frailty assessment tools. As an example of an ultimately short assessment appears the “Clinical Frailty Scale”, in which the assessor chooses from a pictogram with seven to nine graduations at a glance a summary evaluation of “very fit” to “terminally ill” [4]. An evaluation of the given instruments’ applicability and test economy as well as differentiated recommendations for their time requirements is largely lacking. The AGAST (“Association of the Scientific Medical Societies in Germany”) guidelines schedule an estimated CGA duration of 30–35 min [11]. In the range of such geriatric assessment tools with highly variable degrees of complexity the MPI is conducted in a remarkably short period of time, making it a possible target for routine assessment. Investigation and evaluation awaits further, especially head-to-head studies of given CGA tools.

Additionally, the current study may indicate a corresponding learning curve of the single investigator as tnpMPI reached an optimum low of 10 min (as median) in the last visited cohort. As we found a negative association for tnpMPI and the date of the interview (*r* = − 0.559; *p* < 0.01) reflecting the general decrease of tnpMPI in the course of the study, a training effect might be assumed. However, such a training effect or learning curve has not finally been proven, since only a limited number of covariates have been excluded for potential interference: age, gender, number of drugs, polypharmacy and other, overall seven MPI domains. As a matter of fact, additional potential patient- or investigator-related or environmental confounding factors, not recorded by the present study, would have to be considered to demonstrate a training effect. Certainly, the proof of such an effect would require a special study design. Nevertheless, an assessor training in frailty measurement seems to be reasonable with future development of appropriate, more structured instruction and schooling systems in the CGA field [12]. Given an optimum low assessment time of 10 min (median) under our conditions (cohort 3), the MPI positions even more favorable in the ranking position of CGA tools with regard to time expenditure.

Under economic considerations, the question arises which part of CGA information could be generated outside a patient interview. This approach might be helpful to save limited and precious resources, i.e., medical staff and the time factor. As a future perspective, digital data collection and even more artificial intelligence could contribute to facilitate the performance of CGA alike MPI by computerized in-feeding of necessary information concerning diagnoses, number of medications, ADL, IADL, etc. [13]. This may function especially at the checkpoint of hospital admission by means of electronic health card systems as being under most recent development. This logistical point, which will have to be carefully counter-balanced with all aspects of data safety, offers opportunities in regular implementation of CGA in everyday clinical practice [14]. Another future option may be the establishment of a validated patient self-reported CGA tool inaugurated exemplarily as “SELFYMPI” by the working group of A. Pilotto 2019 [15].

The present study has important limitations. Since merely patients hospitalized in geriatric departments were included, the results may not be directly applicable to ambulatory or emergency care patients. However, patients in geriatric outpatient settings might be less complex to evaluate than those included in the present study. Additionally, patients unable to give informed consent themselves (e. g., in case of advanced dementia) had to be excluded from study participation according to the study protocol. This may have led to a certain bias presumably influencing the central study parameter tnpMPI. Moreover, in the follow-up of the patients there was a rather high loss of 40%. This, in combination with the relatively small sample size, may explain that the MPI did not turn out to demonstrate its prognostic value.

In conclusion, in the course of the current study, MPI was recorded in three cohorts of *n* = 30 patients, each visited en bloc sequentially. In the last cohort, the time needed to perform MPI was at an optimum low median of 10 min. Thus, MPI may be used in an adequately practicable, relatively short time frame for the purpose of geriatric assessment. Prospective controlled comparative studies are needed in order to address the question as to whether a single CGA will be established as a gold standard, providing an optimum ratio of predictive value and time expenditure for determination.

## Supplementary Information

Below is the link to the electronic supplementary material.

Supplementary file1 (DOCX 18 kb)

## Data Availability

Data are available upon request.
