# Supplementary material for: The multidimensional prognostic index in hospitalized older adults: practicability with regard to time needs
Source: Aging Clin Exp Res. 2023 Jan 30;35(3):711–6. doi: 10.1007/s40520-022-02311-9 (PMC10014668; doi:10.1007/s40520-022-02311-9)
Supplement: Supplementary file 1 — Supplementary file1 (DOCX 18 kb) [file 40520_2022_2311_MOESM1_ESM.docx]

**Supplementary Table 1**: Analysis of study population according to cohort assignment (descriptive statistics).

| **Condition** | **Total**  n = 90 | **Cohort 1**  n = 30 | **Cohort 2**  n = 30 | **Cohort 3**  n = 30 | ***p*-value** | ***post-hoc-*test** |
| --- | --- | --- | --- | --- | --- | --- |
| **Gender**, female [vs. male]; n (%) | 65 (72.2) | 22 (73.3) | 23 (76.7) | 20 (66.7) | 0.898 | n.d. |
| **Age** (years), median (IQR) | 83.5 (7.0) | 84 (7.5) | 82.5 (6.0) | 84.5 (7.0) | 0.843 | n.d. |
| **Number of diagnoses**, median (IQR) | 6 (4-9) | 6 (4-9) | 4 (3-6) | 8 (7-10) | < 0.001* | cohort 3 > 2, cohort 3 > 1 |
| **Polypharmacy**, n (%) | 49 (54.4) | 15 (50.0) | 16 (53.3) | 18 (60.0) | 0.733 | n.d. |
| **MPI**, median (IQR)  **MPI domains**,  median (IQR): | 0.38 (0.31-0.56) | 0.38 (0.31-0.56) | 0.38 (0.3-0.52) | 0.47 (0.37-0.56) | 0.113 | n.d. |
| CIRS | 1.0 (1.0-2.0) | 1.0 (0.0-2.0) | 1.0 (0.0-2.0) | 2.0 (1.0-3.0) | 0.044 | n.s. |
| ADL | 5.0 (3.0-5.0) | 5.0 (3.0-6.0) | 5.0 (3.0-6.0) | 5.0 (3.0-6.0) | 0.858 | n.d. |
| IADL | 5.0 (3.0-6.0) | 6.0 (3.0-7.0) | 6.0 (4.0-7.0) | 5.0 (3.75-7.0) | 0.792 | n.d. |
| MNA-SF | 6.0 (6.0-8.3) | 6.5 (6.0-8.0) | 7.5 (6.0-9.0) | 6.0 (5.0-8.0) | 0.189 | n.d. |
| SPMSQ (categorized) | 0.0 (0.0-0.0) | 0.0 (0.0-0.0) | 0.0 (0.0-0.0) | 0.0 (0.0-0.0) | 0.163 | n.d. |
| ESS | 16.0  (14.0-17.0) | 16.0  (13.75-17.0) | 16.0  (14.0-17.0) | 16.0  (15.0-17.0) | 0.837 | n.d. |
| Number of drugs | 6.2 (5.0-8.0) | 5.5 (4.0-6.3) | 6.0 (5.0-7.0) | 5.0 (5.0-10.0) | 0.186 | n.d. |
| **Cohabitation status**, n (%): |  |  |  |  | 0.23 | n.d. |
| - with family members | 30 (33.3) | 11 (36.7) | 13 (43.3) | 6 (20.0) |  |  |
| - professional care | 7 (7.8) | 4 (13.3) | 0 (0) | 3 (10.0) |  |  |
| - alone | 53 (58.9) | 15 (50.0) | 17 (56.7) | 21 (70.0) |  |  |
| Total collective of patients comprising cohorts 1 to 3, each visited *en bloc* sequentially in three geriatric units; n = number of patients; IQR = inter quartile range; MPI = Multidimensional Prognostic Index, CIRS = Cumulative Index Rating Scale; ADL = Activities of Daily Living; IADL = Instrumental Activities of Daily Living; MNA-SF = Mini Nutritional Assessment - Short Form; SPMSQ = Short Portable Mental Status Questionnaire; ESS = Exton-Smith-Scale; n.d. = not done, n.s.= not significant, Kruskal-Wallis-test, level of significance < 0.05, * = statistically significant | | | | | | |
